# Treatment of Psychogenic Polydipsia and Hyponatremia: A Case Report

**DOI:** 10.7759/cureus.47719

**Published:** 2023-10-26

**Authors:** Alyse A Hurwit, Jonathan M Parker, Stepan Uhlyar

**Affiliations:** 1 Psychiatry and Behavioral Sciences, Ross University School of Medicine, Miami, USA; 2 Psychiatry and Behavioral Sciences, Jackson Behavioral Health Hospital, Miami, USA; 3 Psychiatry and Behavioral Sciences, University of Miami Health System, Miami, USA; 4 Psychopharmacology, Jackson Behavioral Health Hospital, Miami, USA

**Keywords:** water intoxication, hyponatremia, psychosis, psychogenic polydipsia, treatment

## Abstract

Psychogenic polydipsia occurs during water or fluid intoxication and can lead to electrolyte disturbances, such as hyponatremia. Hyponatremia can give rise to signs and symptoms, including lethargy, psychosis, seizures, or death. Psychogenic, or primary polydipsia, can be compared to other medical conditions that cause excessive thirst. This case report will focus on the symptoms, disease, and treatment involved in the care and hospitalization of a 30-year-old male patient who reported ingesting up to 40 liters of water a day for the last three years. This patient with psychogenic polydipsia, chronic schizophrenia, and active psychosis was diagnosed with metabolic encephalopathy secondary to severe hyponatremia (day one sodium level: 108 mEq/L). The management goal was to stabilize electrolytes and increase sodium levels without causing osmotic demyelination syndrome. During subsequent hospitalization, the psychiatry team worked towards the normalization of sodium levels and managed behavioral patterns contributing to water consumption. The patient achieved a normal sodium level on day 21 of inpatient psychiatric treatment with the following medication regimen: acetazolamide, candesartan, olanzapine, sodium chloride, and trazodone.

## Introduction

The average adult is recommended to ingest 2-4 liters of water a day to be considered adequately hydrated [1]. Polydipsia is described as ingesting over 6 liters of fluid per day. Patients with psychogenic polydipsia are inclined toward compulsive behaviors, such as excessive water intake, which can be a complication of neuropsychiatric disorders. Although it is rare, this condition can be observed in individuals with pre-existing psychiatric disorders. The primary symptom arising from this condition is due to hyponatremia. Sodium levels categorize the severity of hyponatremia: 130-134 mEq/L is considered mild, 125-129 mEq/L is moderate, and 125 mEq/L or below is deemed severe hyponatremia [2]. The following can occur in a hyponatremic episode: nausea, vomiting, lethargy, confusion, tremors, dizziness, blurry vision, and osmotic demyelination convulsions, amongst many more. The diagnostic criteria of psychogenic polydipsia can be met by ruling out other diseases such as diabetes mellitus, dipsogenic diabetes insipidus, and syndrome of inappropriate anti-diuretic hormone (ADH) secretion. Acute hyponatremia can lead to psychotic episodes, brain damage, cerebral edema, coma, and death [3]. It is hypothesized that superfluous water in the body causes the failure of the kidneys to excrete the fluid and causes ADH to rise from the posterior pituitary, signaling the brain to feel increasingly thirsty [4]. We present a case of a patient who survived severe hyponatremia without any significant CT changes in the brain.

## Case presentation

This case depicts a 30-year-old male patient with a past medical history of schizophrenia and cannabis use who presented to the ED with complaints of confusion, headaches, and palpitations. He was found stumbling on the streets with altered mental status and agitation. He arrived with a scalp laceration but could not remember how he obtained it. Upon initial assessment in the ED, his sodium level was 108 mEq/L. The compulsion to drink a large volume of water was found upon history taking and by his repeatedly asking for more water. The patient was transferred to the medical ICU, where he was seen by multiple providers and diagnosed with metabolic encephalopathy. A brain CT scan was performed and demonstrated a right parietal subgaleal hematoma associated with the laceration and no evidence of hydrocephalus, large vascular territorial infarcts, or an intracranial hemorrhage. Rapid brain swelling can occur in settings of acute hyponatremia; however, there was no cerebral edema. Chest X-ray, ECG, and urine toxicology screening for drugs and alcohol were negative. On admission to the psychiatric unit, the patient was combative, disoriented to time and place, uncooperative, and had poor insight. He did not understand the severity of his condition or how his actions contributed to his hospitalization. On evaluation by providers, prominent features of psychosis were noted, including agitation and uneasiness around others. The patient's family provided collateral, indicating that his behavior had been erratic for several months with multiple exacerbations of "urges" to drink fluids. The patient informed us about drinking large quantities of water to help relieve frequent frontal headaches. Episodes of palpitations would sometimes arise with the headaches. Although not clinically apparent on imaging, the medical team suspected the cause of the headaches described was attributable to hyponatremia, resulting in increased intracranial pressure. Prior to hospitalization, the patient refilled liter-sized bottles and, at times, ingested up to 40 L of water daily. During his stay, he was on a water restriction of 1.5-2 L/day, which increased to around 3 L/day when his sodium levels normalized. He denied symptoms of anxiety, depression, suicidal and homicidal ideations, delusions, auditory hallucinations, visual hallucinations, and mania. After consulting the patient's family for collateral information, the medical team learned about the patient's non-compliance with his prescribed anti-psychotic medication, risperidone (Risperdal), at a dose of 2 mg orally daily. At the medical ICU, he was started on haloperidol (Haldol) 5 mg liquid daily for the initial management of psychosis. Upon admission to the inpatient psychiatry unit, this was subsequently changed to risperidone 3 mg oral tablet due to the positive response he had to it on previous admissions. Due to the degree of remaining disorganization, such as unpredictable agitation, self-contradictory behavior, and compulsions to drink water, risperidone was shown to be ineffective and was discontinued. Research indicates that olanzapine (Zyprexa) often achieves better patient adherence than some other medications. Consequently, olanzapine was initiated at a daily dose of 20 mg and adjusted to find the most effective dose to manage his behavior. After medical stabilization and improvement of sodium levels, the patient was able to elaborate on his thoughts about water intoxication. The patient was hyperfocused on drinking as much water as possible to "fix" the chronic frontal headaches he was reporting. During the history-taking process, the patient mentioned experiencing headaches for several years, though he was uncertain about their cause. He expressed feeling "urges" to drink but clarified that there was no internal stimulus compelling him to do so. Table 1 represents the patient's thirst urges during hospitalization, with 10 being the maximum urge to drink.

**Table 1:** Thirst rating scale.

Day of Hospitalization	Thirst Rating
Day 1	10/10
Day 3	10/10
Day 6	10/10
Day 9	6/10
Day 12	5/10
Day 15	7/10
Day 18	7/10
Day 21	6/10

His lack of insight interfered with the treatment goal of dispensing medication to control electrolyte levels and psychotic symptoms. He believed treatment was not necessary and that he was at ease without it. The nephrology team aided in medically managing this patient, and after a consultation, it was noted that this patient had dilute urine, evident by a urine specific gravity of less than 1.005. Table 2 represents the medications prescribed by the patient's psychiatrist and clinical pharmacist used in the treatment of both hyponatremia and psychogenic polydipsia.

**Table 2:** Hyponatremia and psychogenic polydipsia management.

Medication	Dosage	Route of Administration	Usage
Acetazolamide	250 mg twice a day	Oral	Vasopressin antagonist-like effect to correct sodium levels
Candesartan	8 mg daily	Oral	Polydipsia
Olanzapine	20 mg nightly	Oral	Schizophrenia and psychosis
Sodium Chloride	1 g three times a day	Oral	Stabilize serum sodium levels
Trazodone	100 mg nightly as needed	Oral	Insomnia

Acetazolamide was given for the vasopressin antagonist-like effect to correct sodium levels. Candesartan was administered for the polydipsia. Olanzapine was for schizophrenia and psychosis. The sodium chloride tablets helped reduce the patient's urge to drink water, stabilized serum sodium levels and decreased his headaches. Trazodone was dispensed as needed for insomnia. The patient was alert and did not experience double vision, muscle weakness, or difficulty breathing. He experienced neither a loss of consciousness nor episodes suggestive of seizures. Additionally, he did not have any urinary incontinence or signs of renal failure, which are often associated with water intoxication. On imaging, there was no bladder dilation or hydronephrosis. The patient's sodium levels improved from 108 mEq/L to 141 mEq/L during his hospitalization course of 21 days. A serum osmolality was ordered to rule out hypertonic hyponatremia and isotonic hyponatremia (pseudohyponatremia), which can be caused by hyperglycemia or elevated lipids. Table 3 depicts the basic metabolic panel results during the course of his treatment. The blood work for the days not included in the table slightly increased or decreased from the previous days, although not significant enough to include in the data analysis below. 

**Table 3:** Basic metabolic panel. Bolded: Significant abnormal values.

	Day 1	Day 6	Day 9	Day 21
Glucose (mg/dL)	112	85	90	88
Sodium (mEq/L)	108	138	138	141
Potassium (mEq/L)	4.3	4.5	4.9	4.5
Chloride (mEq/L)	82	99	103	111
Pco_2 _(mmHg)	15	28	24	23
Osmolality (mOsmol/kg H_2_O)	219	274	276	283
Anion Gap (mEq/L)	11	11	11	7
BUN (mg/dL)	8	8	15	21
Creatinine (mg/dL)	0.50	0.80	0.80	1.10

## Discussion

Psychogenic polydipsia falls under the classification of primary polydipsia and is found in roughly 20% of patients with psychiatric disorders. The relation to schizophrenia is unknown, but statistics show an increased incidence in this population over schizoaffective disorder and bipolar disorder [5]. Primary polydipsia is caused by unwarranted fluid intake without an underlying cause, and secondary polydipsia can be medication-induced (i.e., from diuretics or laxatives) or due to a medical condition [4]. Diabetes mellitus, dipsogenic diabetes insipidus (DDI), and syndrome of inappropriate anti-diuretic hormone secretion (SIADH) can present with similar manifestations as psychogenic polydipsia. Table 4 displays diseases that cause primary and secondary polydipsia and should be ruled out when considering a treatment plan for psychogenic polydipsia.

**Table 4:** Differential diagnoses: Polydipsia. SIADH: Syndrome of Inappropriate Antidiuretic Hormone Secretion; ADH: Anti-diuretic hormone; TSH: Thyroid-stimulating hormone. Source: [5-8]

Disease	Type of Polydipsia	Epidemiology	Manifestations	Diagnostic Criteria
Psychogenic Polydipsia [5]	Primary	~20% of psychiatric patients	Behavioral, excessive thirst despite the body not needing any more water	Underlying psychiatric illness, serum electrolytes, comprehensive metabolic panel (CMP), urine osmolality test, toxicology screening
Diabetes Mellitus (Type I or II)	Secondary	~11% of the USA population (majority II) [6]	Fatigue, polyuria, polydipsia, weight loss (I), weight gain (II), dry mouth, blurry vision, peripheral neuropathy, recurrent infections	Fasting Blood Glucose, Urinalysis
Dipsogenic Diabetes Insipidus (DDI) [7]	Primary	Rare, ~0.004% globally [8]	Polyuria, polydipsia, diluted urine (light color)	ADH level, water deprivation test, underlying psychiatric illness, MRI (damage to hypothalamus by trauma, infection, tumor, or inflammation)
SIADH	Secondary	Common, exact prevalence is unknown	Tremor, muscle cramps, nausea, depression, seizures, memory deficits, vomiting, confusion	Urinalysis, serum electrolyte levels, serum and urine osmolality test, toxicology screening (medications), and TSH level.

This medical management plan was successful in treating this schizophrenic patient who presented with encephalopathy due to severe hyponatremia in the context of psychogenic polydipsia. Olanzapine is an atypical (2nd generation) antipsychotic with a mechanism that acts as an antagonist on dopamine (D2) and serotonin receptors in the mesolimbic pathway. It is commonly used to treat patients with schizophrenia and decrease polydipsia [9]. Acetazolamide is a diuretic that works by inhibiting carbonic anhydrase on the proximal tubule of the nephron. It reabsorbs sodium, chloride, and bicarbonate and can be an alternative to vasopressin antagonists to treat polydipsia [10]. Candesartan, an angiotensin-II receptor blocker, is commonly used to treat hypertension. Research indicates that candesartan can help improve polydipsia by reducing the urge to drink water, owing to the effects of angiotensin-II on the brain [11]. Sodium chloride tablets help to correct serum sodium levels in euvolemic or hypovolemic hyponatremic patients. However, care must be exercised when adjusting sodium levels in acute situations. Rapid correction can lead to central pontine myelinolysis, also known as osmotic demyelination syndrome, which can cause complete paralysis of voluntary muscles (except in the eye muscles). This could lead to a coma-like state or "locked-in syndrome." To prevent this, sodium levels should be increased by no more than 6 to 12 mEq/L on the first day and less than 18 mEq/L over the course of two days [12-13]. Adverse effects noted by the patient included palpitations and fatigue. He was also restricted to about 3.0 L of water per day. He did not have open access to water and was given a bottle that only staff could refill. With a sodium level of 108 mEq/L, a person could be comatose or expire from cerebral edema and central nervous system dysfunction. Aside from pharmacological management, water restriction was significant in our treatment plan to prevent further abnormalities in lab work and help the patient adjust to a new routine. Pathological water drinking causes a significant dilution of the blood. The kidneys cannot compensate efficiently due to the amount of water ingested. In normal functioning kidneys, vasopressin will be decreased to excrete free water in the urine. Medication compliance in our patient with psychogenic polydipsia was a concern due to his lack of insight, poor judgment, and psychiatric history. With encouragement, he was cooperative in taking oral medication. In patients with psychogenic polydipsia, adherence to the treatment regimen is absolutely necessary to prevent both compulsions to drink excessive fluids as well as the lethal consequences of resulting hyponatremia.

## Conclusions

Consuming 40 liters of water in most individuals would result in drastic and potentially catastrophic effects, likely causing mortality. This specific patient received the medical and psychiatric care that was required to discharge him to a less restrictive level of care at a residential program. Episodes of hyponatremia can be prevented if patients adhere to their prescribed medication regimen in an outpatient setting.
